# Intraperitoneal delivery of acetate-encapsulated liposomal nanoparticles for neuroprotection of the penumbra in a rat model of ischemic stroke

**DOI:** 10.2147/IJN.S193965

**Published:** 2019-03-18

**Authors:** Po-Wah So, Antigoni Ekonomou, Kim Galley, Leigh Brody, Meliz Sahuri-Arisoylu, Ivan Rattray, Diana Cash, Jimmy D Bell

**Affiliations:** 1 King’s College London, Institute of Psychiatry, Psychology and Neuroscience, Department of Neuroimaging, London, UK, po-wah.so@kcl.ac.uk; 2 University of Westminster, Research Centre for Optimal Health, London, UK; 3 King’s College London, Institute of Psychiatry, Psychology and Neuroscience, Department of Basic and Clinical Neuroscience, London, UK

**Keywords:** ischemic stroke, acetate, liposomes, neuroinflammation, microglia, mid-cerebral artery occlusion

## Abstract

**Background:**

Ischemic stroke is a devastating condition, with metabolic derangement and persistent inflammation enhancing the initial insult of ischaemia. Recombinant tissue plasminogen remains the only effective treatment but limited as therapy must commence soon after the onset of symptoms.

**Purpose:**

We investigated whether acetate, which modulates many pathways including inflammation, may attenuate brain injury in stroke. As acetate has a short blood half-life and high amounts irritate the gastrointestinal tract, acetate was administered encapsulated in a liposomal nanoparticle (liposomal-encapsulated acetate, LITA).

**Methods:**

Transient ischemia was induced by 90 mins middle-cerebral artery occlusion (MCAO) in Sprague-Dawley rats, and LITA or control liposomes given intraperitoneally at occlusion and daily for up to two weeks post-MCAO. Magnetic resonance imaging (MRI) was used to estimate lesion volume at 24 h, 1 and 2 weeks post-MCAO and anterior lateral ventricular volume (ALVv) at 2 weeks post-MCAO. Locomotive behaviour was tested prior to the final MRI scan. After the final scan, brains were collected, and immunohistochemistry was performed.

**Results:**

Lesion volumes were decreased by ~80% from 24 h to one-week post-MCAO, in both control and LITA groups (*P*⩽0.05). However, the lesion was increased by ~50% over the subsequent 1 to 2 weeks after MCAO in the control group (from 24.1±10.0 to 58.7±28.6 mm^3^; *P*⩽0.05) but remained unchanged in the LITA group. ALVv were also attenuated by LITA treatment at 2 weeks post-MCAO (177.2±11.9% and 135.3±10.9% of contralateral ALVv for control and LITA groups, respectively; *P*⩽0.05). LITA-treated animals also appeared to have improved motor activity, moving with greater average velocity than control animals. Microglial immunoreactivity was ~40% lower in the LITA group compared to the control group (*P*⩽0.05), but LITA did not modulate neurogenesis, apoptosis, histone acetylation and lipid peroxidation.

**Conclusion:**

LITA appears to attenuate the harmful chronic neuroinflammation observed during brain remodeling after a focal ischemic insult.

## Introduction

Ischemic stroke is caused by obstruction of an artery to the brain from in situ thrombus, or an embolus from another artery or the heart, and one of the main causes of disability in industrialized countries.^1^ The use of recombinant tissue plasminogen activator (rtPA) that breaks down blood clots has significantly improved the morbidity of ischemic stroke.^2^ However, its use remains limited, as rtPA must be administered within 4.5 hours of symptoms and so only 3%–8% of all patients are eligible for therapy.^3^,^4^ In addition, thrombolytic treatment may lead to death due to intracranial hemorrhage.^5^

On induction of ischemia, the sudden lack of oxygen and glucose elicits a cascade of pathological events including abnormal recruitment of inflammatory cells, excess production of free radicals, initiation of pathological apoptosis, excessive glutamate excitotoxicity, and ion dyshomeostasis.^6^ Many of the pathological processes are mediated by mitochondrial dysfunction including increased lipid peroxidation leading to oxidative stress and apoptosis.^7^

Rapid neuronal death ensues in the immediate vicinity of ischemia, but around this initial infarcted area or ischemic core is the penumbra which is hypo-perfused with collateral blood flow maintaining some cellular functions. The penumbra is not passively dying over time but also actively recovering sometime after induction of ischemic stroke^8^ and the concept of the penumbra lengthens the therapeutic window. Thus, treatments to enhance the brain’s own regenerative capacities, eg, by neurotrophic factors and cell replacement by transplantation have been proposed.^9^

Neuroinflammation progresses from hours to days following induction of ischemic stroke and is characterized by microglial activation and infiltration of circulating inflammatory cells.^10^ Microglia are activated following ischemic stroke, changing shape and phenotype, becoming amoeboid with shorter and thickened processes, and are comparable to macrophages in systemic inflammation. Activated microglia can phagocytose pathogens and necrotic cells, suppress inflammation, and aid in brain repair.^11^ They also produce cytokines, both cytotoxic or cytoprotective,^12^ as well as matrix metalloproteinases (MMPs). MMPs disrupt the blood–brain–barrier (BBB) and allow peripheral leukocytes to infiltrate into the brain, exacerbating neuroinflammation and brain damage.^13^ Thus, systemic inflammatory status, prior to and at the time of stroke, is a major factor determining acute outcome as well as long-term prognosis.^14^

Anti-inflammatory treatments have been shown to reduce infarct size and improve neurological function,^15^ eg, overexpression of the anti-inflammatory cytokine IL-10 and treatment with anti-inflammatory, insulin-like growth factor reduced infarct volumes^16^ and attenuated motor-sensory dysfunction.^17^ Propofol, another anti-inflammatory agent, has been shown to inhibit microglia-mediated inflammation in a rat model of ischemic stroke, again reducing infarct volume and improving neurological functions.^18^ Nafamostat mesilate, commonly used for treating inflammatory diseases such as pancreatitis, has been demonstrated to decrease infarct size and expression of pro-inflammatory mediators and promotes expression of anti-inflammatory molecules in transient middle cerebral artery occlusion (MCAO) in rats.^19^

Short chain fatty acids including acetate have been shown to attenuate inflammation.^20^ To the best of our knowledge, the effects of acetate have not been tested in experimental models of stroke. We hypothesized that acetate supplementation may attenuate the detrimental inflammation following induction of ischemia and protect the brain from further damage after the initial insult. However, while acetate in the form of calcium acetate is approved to control high blood phosphate levels in kidney disease,^21^ its half-life is short and induces gastrointestinal irritation.^22^ Thus, we propose the administration of acetate encapsulated within a liposome nanoparticle, liposome-encapsulated acetate (LITA) to extend the half-life of acetate, as well as achieve a high payload with minimum toxicity.^23^ LITA has a polyethyleneglycol (PEG) coating to attenuate its clearance from the systemic circulation by the reticuloendothelial system.^24^ Furthermore, encapsulation of therapeutic compounds in liposomes has been shown to enhance therapeutic effects of drugs by providing an “inert” local environment to maintain their stability as well as prolong their blood residence time and thereby increase bioavailability to brain tissue.^25^,^26^

In this study, we assessed if administration of LITA attenuates brain injury following ischemic stroke induced by MCAO. We performed MRI to longitudinally assess infarct volume at 24 hours, 1 and 2 weeks post-MCAO: T2-weighted MRI is commonly used to serially assess infarct size.^27^ Locomotion in an open arena was also assessed as a surrogate measure of recovery prior to the last MRI scan. After the final scan, brains were harvested, and immunohistochemistry was performed to evaluate mitochondria, neurogenesis, apoptosis, and lipid peroxidation to assess the effects of acetate pathological processes in stroke. Histone H3 acetylation was also measured as acetate action may be mediated via epigenetic modulation by acetate acetylation of histones.

## Materials and methods

### Animals and treatment

For biodistribution studies (In vivo distribution of liposomal nanoparticles section) of LITA, male C57Bl/6J mice (8–10 weeks old, n=8; Harlan, UK) were obtained and allowed to acclimatize for 5–7 days prior to intravenous (iv) injection with 10 MBq [^18^F]fluorodeoxyglucose ([^18^F]FDG) or liposomes encapsulated [^18^F]FDG (n=4/group).

To test the effect of LITA on ischemic stroke, male Sprague Dawley rats (230–250 g, n=20; Harlan) were obtained and allowed to acclimatize for 5–7 days prior to induction of MCAO (Induction of MCAO treatment section). Animals were allocated to two groups with similar body weights to receive LITA or liposome-encapsulated 4-(2-hydroxyethyl)-1-piperazineethanesulfonic acid (HEPES, control).

All experimental procedures on mice and rats were performed with ethical approvals from the local ethical review panels of Imperial College London and King’s College London, respectively, in compliance with the UK Home Office Animals Scientific Procedures Act 1986.

### Liposomal nanoparticles

#### Preparation of liposomes-encapsulated HEPES (control) or LITA

Liposomes were prepared as previously described.^23^ Briefly, liposomes containing N1-cholesteryloxycarbonyl-3, 7-diazanonane-1,9-diamine, 1,2-distearoyl-sn-glycero-3-phosphocholine, cholesterol, 1,2-dioleoyl-sn-glycero-3-phosphoethanolamine-N-(lissamine rhodamine B sulfonyl) (DOPE-Rhodamine), and 1,2-distearoyl-sn-glycero-3-phos-phoethanolamine-N-methoxy-(polyethylene glycol)-2000 (DSPE-PEG2000), were purchased from Sigma-Aldrich Co. (St Louis, MO, USA) or Avanti Polar Lipids, Inc (Alabaster, AL, USA). LITA nanoparticles were prepared by the thin lipid film hydration method and formulated by initial hydration of appropriate thin lipid films with 1 M acetate, pH 2.3. Lipid suspensions were then sonicated (1 hour, 30°C, in the dark) before their pH were adjusted to 7.0 using sodium hydroxide (pH 12.0) or hydrochloric acid (pH 2.0) (Sigma-Aldrich Co.). The final preparations of nanoparticles were obtained following extensive dialysis (Float-A-lyzer G2; Spectrum Labs, Los Angeles, CA, USA) over 18 hours with changes of deionized water every 4–5 hours. Corresponding control (no acetate) liposomes were prepared similarly except thin lipid film hydration was performed using 4 mM HEPES, pH 6.5, 135 mM sodium chloride. Final LITA and control nanoparticle sizes were determined using a Zetasizer (Malvern Instruments, Malvern, UK).

The formulation of control nanoparticles and LITA was chosen based on extensive testing of a variety of differentially formulated liposomes. The aim was to synthesize nanoparticles of good size (100 nm), possess in vitro stability and maximal acetate encapsulation.^23^ We showed acetate was stably encapsulated in LITA for well over a week in vitro,^28^ but we were unable to assess acetate release from LITA in vivo. LITA-type nanoparticles were synthesized encapsulating ^13^C-acetate to enable detection by ^13^C-nuclear magnetic resonance spectroscopy. We were unable to detect liposomal release of ^13^C-acetate into the blood following systemic administration; indeed, we were not even able to detect the equivalent unencapsulated ^13^C-acetate dose (Brody et al, unpublished results). However, this is unsurprising as basal acetate concentrations in rodents (and some humans) are ~100 uM^25^ and our acetate doses are ~4.5 uM. Furthermore, the injected dose would have been diluted in the overall circulation of the body following administration.

Different formulations also produce varying sized liposomes. We aimed to formulate liposomes of ~100 nm, a nanoparticle size previously shown to be optimal for prolonged in vivo circulation of PEGylated liposomes,^28^ to enhance liposomal uptake by the brain. In summary, the final choice of liposomal nanoparticle balanced the optimal size for increased in vivo circulation time while achieving optimal and stable encapsulation of acetate.

#### In vivo biodistribution of liposomal nanoparticles

Real-time biodistribution of the liposomal nanoparticle was assessed by micro positron emission tomography-computerized tomography (PET-CT; Siemens, Forchheim, Germany). Liposomes were formulated as described above but with [^18^F] FDG during hydration of the thin lipid films, ensuring a dose of 10 MBq of [^18^F]FDG encapsulation per 200 µL injection. Mice were anesthetized with 2%–2.5% isoflurane, 1 L/min oxygen and received iv injection of either 10 MBq [^18^F]FDG or liposome-encapsulated-[^18^F]FDG (n=4/group) and PET-CT images acquired for 30 minutes.^26^ PET signal intensities were obtained from an appropriate region of interest (ROI) in the brain at different times after dosing to provide standard uptake value (SUV) curves. The area under the curve (AUC) was calculated from the SUV curves to estimate uptake of [^18^F] FDG and liposome-encapsulated-[^18^F]FDG into the brain.

### Rat model of MCAO

#### Induction of MCAO and treatment

For the induction of MCAO, rats were anesthetized with 2% isoflurane, oxygen–air mix (30:70). A midline incision was made to expose the common carotid artery. A 4-0 monofilament nylon suture (Doccol Corporation, Sharon, MA, USA) with a silicone rubber coated tip (0.33 mm diameter, 4–5 mm long) was introduced into the right external carotid artery and advanced along the inner carotid artery until 10 mm past the carotid canal which insured blockage of the origin of the middle cerebral artery (MCA) as previously described.^29^ An intraperitoneal (ip) injection (1 mL/300 kg body weight) of HEPES encapsulated liposomes (control group) or LITA (264.82 µg/mL, 4.41 mM) was given at occlusion. Animals were then allowed to recover from anesthesia during MCAO. After 90 minutes of MCAO, animals were re-anesthetized, and the filament was retracted completely to allow reperfusion of the MCA. Saline (3 mL) was given subcutaneously to attenuate possible dehydration. Body temperature was maintained using a heating blanket and a rectal probe during surgery.

Animals were also given an ip dose of either control (1 mL/300 kg) or LITA nanoparticles (1 mL/300 kg) daily for up to 2 weeks after MCAO. MRI was performed at 24 hours, 1 and 2 weeks post-MCAO (Magnetic resonance imaging section) to estimate infarct volume as well as the anterior lateral ventricular volume (ALVv) which increases with increasing infarct volume. Locomotion (average velocity and distance traveled) in an open arena (Locomotive behavior section) was assessed as a surrogate measure of motor recovery before the final MRI scan. Body weights were also recorded daily before the surgery (baseline) and then daily for 2 weeks after MCAO.

After the final MRI scan, brains were perfusion-fixed with 4% paraformaldehyde (PFA, PBS), harvested, and immersed in 4% PFA for a week, prior to storage in PBS until placement in 30% sucrose (PBS) for subsequent cryosectioning and immunofluorescence (Immunofluorescence microscopy section). Immunofluorescence was performed using primary antibodies for astrocytes (GFAP), microglia/macrophage (Iba1), mitochondrial density (MTCO1), lipid peroxidation (MDA), general proliferation (Ki67), neurogenesis (nestin), histone modification (acetylated histone H3), and apoptosis and neurodegeneration (appoptosin). (Iba1 does not distinguish between microglia and macrophages infiltrating the brain from the periphery).

### Magnetic resonance imaging

Rats were anaesthetized with isoflurane, oxygen-air (30%:70%) mix. The rat head was carefully located centrally in a 43 mm inner diameter quadrature volume MRI coil and then placed within the magnet bore of a 7T MR scanner (Agilent Technologies, Santa Clara, CA, USA). T2-weighted MRI was performed using a fast spin-echo sequence with repetition time (TR), 4,000 ms; effective echo time (TE), 60 ms; echo train length, 8; four averages; 24 contiguous coronal slices (1 mm thick) were recorded of the whole rat brain with field of view, 32×32 mm and matrix size, 128×128.

Infarct volumes on the T2-weighted MR images were manually segmented using ImageJ (1.46 r, National Institutes of Health (NIH), Bethesda, Maryland, USA) at 24 hours, 1 and 2 weeks after MCAO. The circumference of the ventricle wall on the ipsilateral (stroke side) and contralateral sides were measured from three to five sections per animal at the level of striatum at two weeks after MCAO to provide the ALVv and expressed as a mean percentage of the ipsilateral side to the contralateral one.

### Locomotive behavior

The open field test was performed to evaluate locomotor activity at 10 days after MCAO. Behavioral testing was carried out in a quiet and dimly lit behavior room in white featureless arenas. Rats were individually placed in the arena, and their behavior was recorded for 30 minutes via a video camera positioned on the ceiling, directly above the arenas. The total distance covered by the rat and the average velocity was measured using EthoVision XT software (Noldus, Wageningen, the Netherlands). After the end of the recording, animals were returned to their cages and the arenas were thoroughly cleaned with 70% industrial methylated spirit solution between each set of animals.

### Immunofluorescence microscopy

Coronal brain sections (20 µm thick) were sectioned using a freezing microtome (Carl Zeiss Meditec AG, Jena, Germany), collected onto positively charged slides, and stored at −20°C until use. Three to five sections per animal (spaced 200 µm apart) were used for each antibody, between the levels of the anterior and posterior striatum. Single- and double-immunofluorescence was performed with a few modifications as previously described.^30^ Briefly, sections were incubated in 10% horse serum in Tris-buffered saline (pH 7.4) plus 0.1% Triton X-100 (TBS+) for 2 hours at room temperature prior to incubation in 2% horse serum in TBS+ containing either single or combinations of primary antibodies: rabbit polyclonal anti-Ki67 (clone sp6, 1:200; Thermo Fisher Scientific, Waltham, MA, USA) and mouse monoclonal anti-nestin (rat 401, 1:90; Developmental Studies Hybridoma Bank, Iowa City, IA, USA); rabbit poly-clonal anti-GFAP (1:5,000; Dako Denmark A/S, Glostrup, Denmark) and mouse monoclonal anti-NeuN (1:500; EMD Millipore, Billerica, MA, USA); rabbit polyclonal ionized calcium-binding adaptor (Iba1, 1:1,500; Wako Pure Chemical Industries, Ltd., Osaka, Japan); rabbit polyclonal anti-appoptosin (SLC25A38, 1:250; Sigma-Aldrich Co.); rabbit polyclonal anti-accHH3 (1:300; EMD Millipore); mouse MTCO1 (1:100) and rabbit polyclonal anti-MDA (1:250; both from Abcam, UK) at 4°C overnight. After washes, sections were incubated in the appropriate secondary fluorescent antibodies (1:1,000, Alexa products; Thermo Fisher Scientific) diluted in TBS+ for 2 hours at room temperature. After washes in TBS+ (×2) and TBS–buffers (the former containing Hoescht33258 as a counterstain), slides were mounted using Fluorsave (Calbiochem, UK). For negative control, either the primary or the secondary antibodies were omitted to reveal non-specific labeling.

Mean immunopositive cell numbers (Ki67) and mean percentage of area of immunoreactivity (GFAP, Iba1, nestin) were measured in three separate optical fields per section (×40 magnification) at the peri-infarct area. For appoptosin, MDA, and accHH3, an estimation of the immunoreactivity was performed in the whole of the peri-infarct area by scoring from 0 (no immunoreactivity) to 5 (very high immunoreactivity). Each optical field photo was taken under identical conditions using a Zeiss AxioImager fluorescent microscope (Carl Zeiss Meditec AG) and measurements were performed using the ImageJ software after applying an appropriate threshold level (1.46 r; NIH).

### Statistical analysis

A mixed design ANOVA was applied to determine differences in body weight and infarct size followed by least significant differences post-hoc analysis. Differences in the immunofluorescence and ALVv data between the groups receiving control or LITA were tested using Student’s unpaired *t*-test. Multiple regression analysis was also applied with treatment group and infarct size at 24 hours after MCAO as independent variables, to determine predictors of body weight, immunofluorescence findings, behavior, and percentage changes in infarct size (24 hours to 1 week and 1–2 weeks after MCAO) and percentage ALVv at 2 weeks post-MCAO). Differences were considered significant if *P*⩽0.05, and IBM SPSS software (version 21) was used for all testing. All values presented are mean ± standard error of mean (SEM).

## Results

### Biodistribution of liposomal nanoparticle

**Figure 1:**
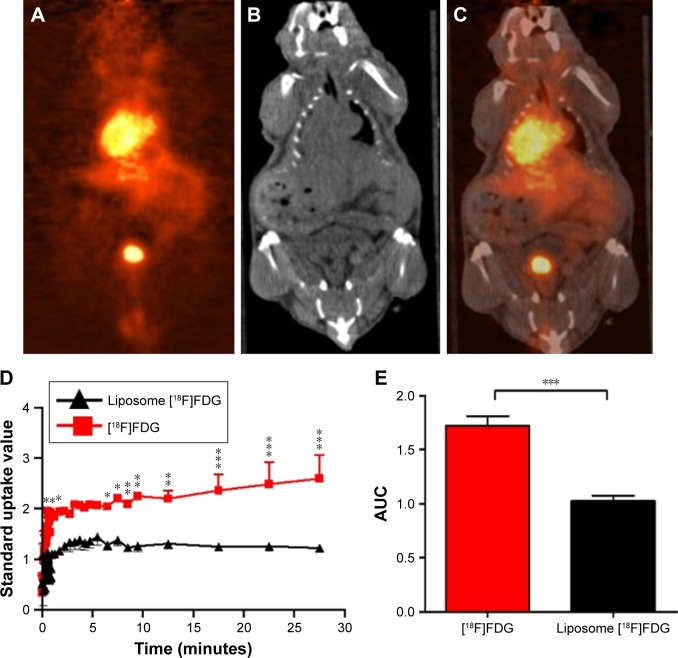
Biodistribution of liposomal nanoparticles. **Notes:** Representative (**A**) positron emission tomography (PET); (**B**) computerized tomography (CT); (**C**) PET-CT fusion images at 30 minutes after an intravenous (iv) injection of 200 µL liposome-encapsulated [^18^F]fluorodeoxyglucose ([^18^F]FDG) in normal mice; (**D**) brain standard uptake value (SUV) curves measured for 30 minutes after iv injection of [^18^F]FDG or liposome-encapsulated [^18^F]FDG; and (**E**) area under curve (AUC) of brain SUV curves. Values are presented as mean ± standard error of mean. *, **, and *** are *P*⩽0.05, ⩽0.01 and ⩽0.001, respectively.

Control liposomes and LITA were measured to be sized 95.9±9.0 nm and 102.3±7 nm, respectively. In vivo bio-distribution of the liposomal nanoparticle was determined by in vivo PET-CT imaging of [^18^F]FDG-encapsulated in liposomal nanoparticles, injected iv in normal mice. Uptake of the nanoparticle was observed in several organs including the liver, heart, kidneys, and pancreas (Figure 1A–C). While uptake of liposome-encapsulated [^18^F]FDG into the brain was observed, uptake was significantly lower than that for non-encapsulated [^18^F]-FDG (SUV, *P*⩽0.01; AUC, *P*<0.001; Figure 1D and E).

### Body weight

Body weights were similar in the two groups following MCAO, decreasing rapidly and remaining low for approximately a week before steadily increasing (Figure S1). Treatment group and lesion volume at 24 hours after MCAO statistically significantly predicted the body weight at 2 weeks after MCAO (F(2,14)=5.798, *P*=0.015, R^2^=0.375; Table 1), but only lesion volume at 24 hours significantly contributed to the prediction (*P*=0.004, Table 1).

### Regional brain volumes by MRI

#### Infarct volume

**Figure 2:**
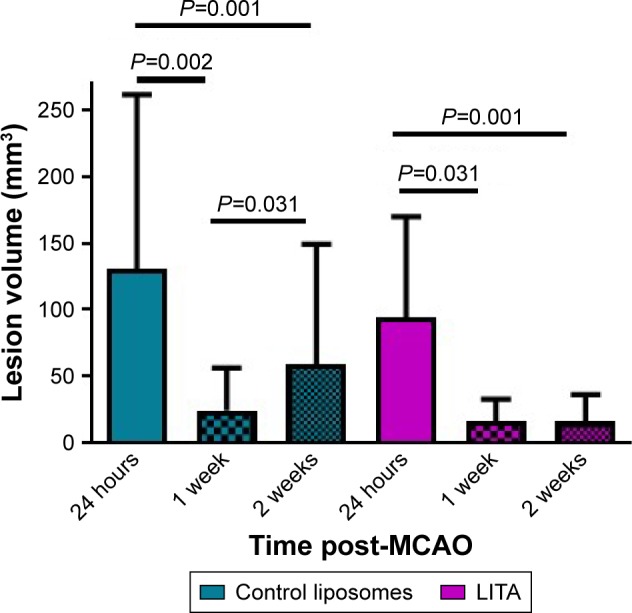
MRI assessment of infarct volumes in rats treated with control or liposomal encapsulated acetate (LITA) at 24 hours, 1, and 2 weeks after middle-cerebral artery occlusion (MCAO). **Note:** Values are presented as mean ± standard error of mean.

MCAO led to infarct in the striatal-cortical region and appeared hyperintense with occasional small hypointense areas from necrosis by MRI (Figure S2). Infarct volumes were reduced by 85% and 83% in the first week after MCAO in control and LITA groups, respectively (*P*=0.002 and 0.031, respectively; Figure 2). Similarly, infarct volumes remained lower at 2 weeks compared to that at 24 hours after MCAO for both control and LITA groups (55% and 83% reduction, both *P*=0.001). However, while the infarct volume increased by 24.3% over 1–2 weeks after MCAO in the control group (*P*=0.031, Figure 2), the infarct volume during this time were essentially unchanged in the LITA group (*P*=0.982, Figure 2).

Treatment group and infarct size at 24 hours after MCAO significantly predicted the percentage change in infarct volume over 1–2 weeks after MCAO (F(2,14)=35.964, *P*=3×10-6, R^2^=0.814; Table 2). Both LITA treatment and the infarct volume at 24 hours after MCAO added significantly to the prediction (*P*=0.049 and *P*=2×10^–7^, respectively; Table 1).

#### Anterior lateral ventricular volume

Expansion of the ALVv ipsilateral to the stroke arises from the shrinkage of the striatal-cortical areas due to infarction and so can be considered a measure of brain damage. The percentage of the ALVv ipsilateral to the stroke lesion relative to the contralateral ALVv was significantly higher in the control compared to the LITA group at 2 weeks post-MCAO (177.2%±11.9% and 135.3%±10.9%, respectively, *P*=0.020; Table 2). Both treatment group and infarct volume at 24 hours post-MCAO predicted the increase in ALV ipsilateral to the stroke (F(2,14)=6.068, *P*=0.013, R^2^=0.388; Table 2) but only the treatment group added significantly to the prediction (*P*=0.033).

### Locomotive behavior

**Figure 3:**
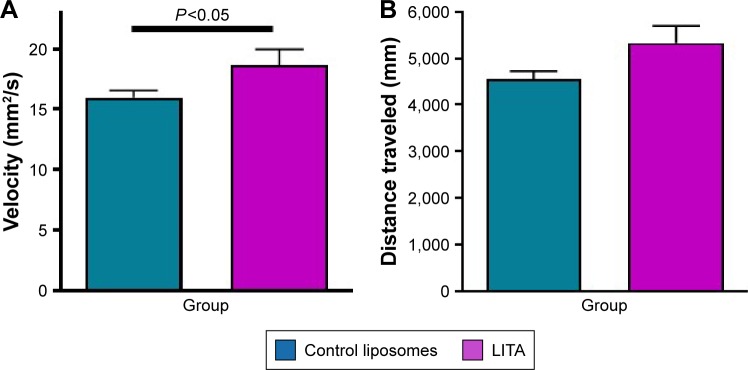
Locomotive behavior: (**A**) average velocity and (**B**) distance traveled of rats treated with control or liposomal encapsulated acetate (LITA) at 10 days after middle-cerebral artery occlusion. Values are mean ± standard error of mean.

In open field behavior testing, the LITA group moved at greater velocity (17%) than those in the control group (*P*⩽0.05; Figure 3A; Table 2). The former also traveled greater distances than the latter, although significance was not quite reached (Figure 3B; 17%, *P*=0.054). Multiple regression analysis revealed that the prediction of motor recovery almost reached significance for the treatment group (*P*=0.057 and 0.059 for velocity and distance traveled; Table 1).

### Immunofluorescence

#### Neuroinflammation

**Figure 4:**
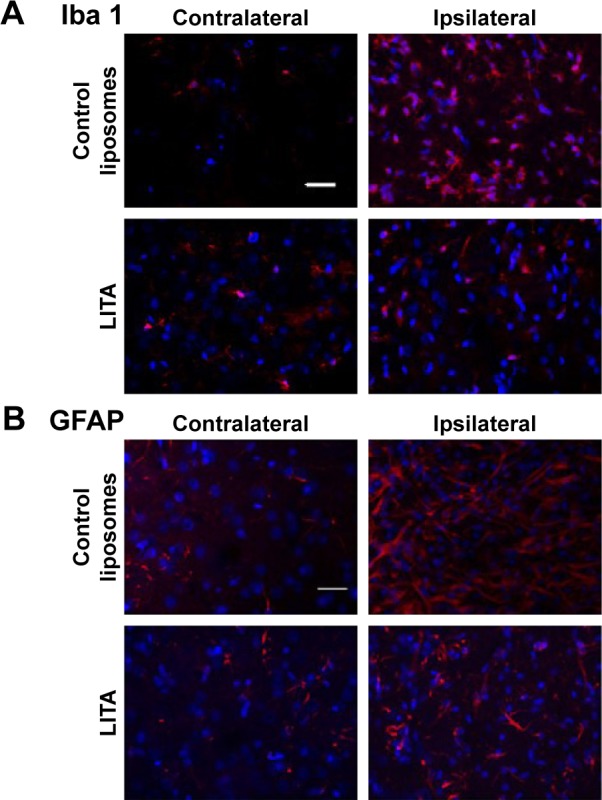
Immunofluorescence micrographs of (**A**) Iba 1 and (**B**) GFAP immunoreactivities in the peri-infarct area (ipsilateral to the stroke lesion) and the corresponding region in the contralateral hemisphere in control or liposomal encapsulated acetate (LITA)-treated animals at 2 weeks after middle-cerebral artery occlusion (scale bar: 50 µm).

Neuroinflammation at 2 weeks after MCAO was assessed by immunofluorescence using antibodies against Iba1 and GFAP to visualize microglia/macrophages and astrocytes, respectively, which are increased/activated during neuroinflammation (Figure 4A and B). Percentage microglia/macrophages immunoreactivity 2 weeks after MCAO was lower in the LITA group compared to the control group (38.18%, *P*=0.031; Table 2). Furthermore, treatment and infarct volume at 24 hours after MCAO significantly predicted the change in Iba1 immunoreactivity in the ipsilateral region to the stroke (F(2,12)=5.355, *P*=0.022, R^2^=0.384; Table 1), although neither factor added significantly to the prediction. Astrocytosis appeared similar in both control and LITA groups (Table 2), although there was a trend of lower percentage GFAP immunoreactivity in the peri-infarct region in the latter animals (Figure 4B). Treatment group and infarct volume at 24 hours after MCAO did not predict the changes in astrocytosis at 2 weeks after MCAO (Table 1). Neuroinflammation at 2 weeks after MCAO appeared to be mediated by microglia rather than astrocytes in the transient ischaemia MCAO rat model.

#### Mitochondrial density

Mitochondrial dysfunction mediates many of the pathological processes during ischemic stroke. To assess the effect of LITA on mitochondria, 2 weeks after MCAO, the mitochondria density was estimated in the peri-infarct area using the MTC01 antibody (reacts with cytochrome c oxidase subunit-1, a key mitochondrial enzyme). Mitochondria density was comparable in both control and LITA groups (Table 2; Figure S3) and not predicted by treatment group or infarct volume at 24 hours after MCAO (Table 1).

#### Lipid peroxidation

Following induction of transient ischemia, the restoration of oxygen to the brain leads to elevated ROS and increased lipid peroxidation with the production of molecules such as MDA. MDA immunoreactivity at the peri-infarct area was shown to be similar between control and LITA groups, although generally lower in the latter (Table 2; Figure S3). However, treatment group and infarct volume at 24 hours after MCAO significantly predicted the degree of MDA immunoreactivity in the peri-infarct area (F(2,14)=14.87, *P*=0.001, R^2^=0.665) with the infarct volume at 24 hours after MCAO significantly added to the prediction (*P*=0.0002, Table 1), but not the treatment.

#### Proliferation

Cell proliferation, as assessed by Ki67 immunoreactivity, was similar in both control and LITA groups, although there appeared to be a trend for fewer proliferating cells in the latter (Table 2; Figure S3). Treatment group and infarct volume at 24 hours after MCAO significantly predicted numbers of proliferating cells ipsilateral to the stroke lesion (F(2,12)=9.282, *P*=0.004, R^2^=0.542), with the infarct volume at 24 hours after MCAO statistically significantly adding to the prediction (*P*=0.002; Table 2).

#### Endogenous neurogenesis

Nestin is expressed by neuronal progenitor cells. Nestin immunoreactivity was similar between the control and LITA groups, although a trend to lesser immunoreactivity in the latter was observed (Table 2; Figure S3), suggesting similar neurogenesis between the two groups. The degree of neurogenesis was not predicted by the treatment group or infarct volume at 24 hours after MCAO (Table 1).

#### Histone H3 acetylation

Possible epigenetic modulation arising from LITA was investigated by measuring the degree of histone H3 acetylation. Acetylated histone H3 (accHH3) immunoreactivities were similar between the control and LITA groups at 2 weeks after MCAO (Table 2; Figure S3). However, treatment group and infarct volume at 24 hours after MCAO significantly predicted accHH3 immunoreactivity (F(2,14)=9.136, *P*=0.004, R^2^=0.538), but only the infarct volume statistically significantly contributed to the prediction (*P*=0.001, Table 1).

#### Apoptosis (appoptosin)

Apoptosis (indicated by appoptosin immunoreactivity) in the peri-infarct area was similar in both the control and LITA groups at 2 weeks after MCAO (Table 2; Figure S3) and can be predicted from group and infarct volume at 24 hours post-MCAO (F(2,14)=7.661, *P*=0.007, R^2^=0.488); the infarct volume statistically significantly added to the prediction (*P*=0.002, Table 1).

## Discussion

MCAO leads to irreversible cell damage in the immediate brain area and is known to induce a cascade of pathophysiological processes in the penumbra leading to cell death and increasing infarct volume sometime after the insult.^6^ In our study, LITA appears to protect the penumbra from entering irreversible cell death by preventing further increases in the volumes of the infarct 1–2 weeks after MCAO and the ALVv at 2 weeks post-MCAO. Locomotor behavior testing at 10 days after MCAO suggests greater motor recovery in the LITA group. Neuroprotection of the penumbra by LITA appears to be due to attenuated neuroinflammation mediated by microglia. Lipid per-oxidation, proliferation, endogenous neurogenesis, histone H3 acetylation, and apoptosis were similar between the control and LITA groups but were dependent on the infarct volume 24 hours after MCAO, which was relatively variable between animals in our 90 minutes transient ischemia MCAO rat model.

We have previously shown that LITA nanoparticles can effectively modulate metabolic processes associated with mitochondrial function and lipid metabolism in peripheral healthy tissues and ectopic tumors.^23^,^31^ LITA has been shown to attenuate peripheral inflammatory responses,^23^ and Wang et al^19^ have demonstrated significant contribution of the periphery to MCAO-induced brain damage. Here, we extend this work to show that these nanoparticles can also have beneficial effects on cerebral tissue following MCAO. Cationic liposomes have been shown to enter the brain by absorptive-mediated endocytosis.^32^ Using [^18^F]FDG to estimate the in vivo biodistribution of liposomal payloads, we show that not much of the [^18^F]FDG encapsulated in our liposomal nanoparticles can enter the brain of normal mice. However, induction of MCAO has been shown to significantly disrupt the BBB for up to 4 weeks following reperfusion.^33^ Furthermore, increased BBB permeability has an “enhanced permeability and retention effect”,^34^ similar to that observed in tumors,^31^ during which considerable uptake of LITA into the brain may occur; a recent review reports the brain accumulation of liposomes in experimental stroke models.^35^ In addition, LITA are significantly PEGylated, which prolongs their circulation time, resulting in preferential accumulation at sites of leaky vasculature such as tumors or brain tissue following BBB disruption. We do note that determining nanoparticle distribution by [^18^F]FDG PET could be slightly misleading as [^18^F]FDG may be released from the circulating liposomes. However, using [^18^F]FDG to estimate in vivo distribution of liposomal payloads in normal mice, we show there is little [^18^F]FDG in the brain, consistent with our previous studies using similar, but fluorescent-labeled liposomes.^25^ In addition, we show non-encapsulated [^18^F] FDG better penetrate the brain than encapsulated [^18^F]FDG, suggesting cargo release from liposome nanoparticles to be minimal.

The neuroprotective action of LITA is likely to arise from attenuation of the detrimental effects of the microglia/macrophage activation following MCAO, as LITA did not modulate lipid peroxidation, endogenous neurogenesis, histone H3 acetylation, or apoptosis following ischemic insult. Also, LITA treatment prevented the increase in the infarct volume 1–2 weeks after MCAO, alongside an increase in the ALVv at 2 weeks post-MCAO, suggesting the beneficial effects of LITA are only evident sometime after the initial injury. Thus, LITA may have the advantage over rtPA by being effective after the initial ischemic insult.

Microglia and macrophages exhibit either M1 or M2 phenotypes. M1 microglial/macrophages usually release destructive proinflammatory mediators on stimulation, eg, by lipopolysaccharide (LPS),^36^ whereas the M2 phenotype is activated by, eg, interleukin-4, and possess “neuroprotective” properties including enhanced phagocytic activity and reduced production of inflammatory mediators.^37^ Following induction of 60 minutes transient ischemia by MCAO, Hu et al^37^ showed that the majority of microglia/macrophages in the infarct were of the M2 phenotype, promoting cortical neuron survival. However, within a week of MCAO, the M2 phagocytic response was attenuated and M1 microglia/macrophages dominated the injured area. Concomitant with the significantly lesser microglial/macrophage reaction at two weeks after MCAO in our study with LITA, and the known transition of microglia/macrophage from M2 to M1 phenotype by this time, acetate may be attenuating the detrimental inflammation presided over by the M1 phenotype and protecting the penumbra from damage. We suggest a longer interventional period would be advantageous, considering brain tissue remodeling after stroke can occur for a protracted period of time following the primary ischemic event. Indeed, chronic brain tissue remodeling was evident even a year after a 2-hour transient ischemic insult in a rat model.^38^

The effects of LITA may be mediated by histone acetylation as histone hyper-acetylation has been shown to be anti-inflammatory^39^ and can alter gene expression.^40^ Reisenauer et al^41^ were able to elevate brain acetate levels with oral glyceryl triacetate (GTA) to attenuate neuroglial activation in a model of LPS-induced neuroinflammation.^42^ In normal rats, GTA increased acetylation of certain histones including H4 at lysine 8 but did not acetylate other histones^41^ and reversed the reduction in H3 acetylation in LPS-induced inflammation.^43^ We were unable to detect changes in histone H3 acetylation with LITA treatment, but this may be due to the insensitivity of our immunofluorescence methods and more sensitive Western blotting methods needs to be employed. Furthermore, in our study, acetate (LITA) treatment was relatively short-term.

In the body, acetate can be generated in vivo by gut bacteria, from the digestion of resistant starches.^22^,^44^ Our study suggests that a diet rich in resistant starch or a ketogenic diet may be advantageous following stroke, consistent with previous reports.^45^ Indeed, we have shown that a high resistance starch-containing diet is able to modulate brain function in a normal mouse model.^46^ However, nutritional interventions may be insufficient to maintain adequate/prolonged circulation of short-chain fatty acids to enhance their accumulation in the brain, which clearly can be achieved by encapsulation of acetate in PEGylated liposomes. Furthermore, delivery of acetate in nanoparticles provides the opportunity to modify these delivery agents to enhance brain targeting for therapeutic treatment of neurodegenerative disorders, eg, Alzheimer’s disease (AD), where the BBB is not as obviously disrupted as in our MCAO model.^47^ Inappropriate chronic microglial inflammation is a characteristic feature of many neurodegenerative diseases including AD^30^ and LITA may be a potential therapy for such diseases and further investigations are warranted.

## Conclusion

We have shown that LITA appears to attenuate the harmful chronic neuroinflammation mediated by microglia/macrophages during brain remodeling after a focal ischemic insult.

## Supplementary materials

Figure S1 Daily body weights of rats treated with control and liposome-encapsulated acetate (LITA) during the 2 weeks after middle-cerebral artery occlusion (MCAO).

Figure S2 Typical coronal in vivo T2-weighted magnetic resonance images of the brain at −0.10 Bregma of control and liposomal encapsulated acetate (LITA) treated rats at 2 weeks after middle-cerebral artery occlusion.
**Notes:** White and yellow arrows indicate the infarct area and anterior lateral ventricle, respectively. Scale bar: 3.0 mm.

Figure S3 Immunofluorescence for mitochondrial density (MTCO1), lipid peroxidation (malondialdehyde, MDA), neural progenitors (nestin), proliferation (Ki67), histone H3 acetylation (accHH3), and appoptosis (appoptosin) in control or liposomal encapsulated acetate (LITA)-treated animals at 2 weeks after middle-cerebral artery occlusion.
**Note:** Scale bar: 50 micrometer.

## Figures and Tables

**Table 1:** Multiple regression analysis with treatment group and infarct size at 24 hours after mid-cerebral artery occlusion (MCAO) as independent variables

Parameter (dependent variables)	Test statistic, significance and regression coefficient	Treatment group, *P*-value	Infarct size (24 hours post-MCAO), *P*-value
Body weight (2 weeks MCAO)	F(2,14)=6.117, *P*=0.012^*^, R^2^=0.393	0.803	0.004^**^
**Volumes by MRI**			
Percentage change in infarct volume over 24 hours to 1 week post-MCAO	F(2,14)=4.677, *P*=0.028^*^, R^2^=0.315	0.25	0.001^***^
Percentage change in infarct volume over 1–2 weeks post-MCAO	F(2,14)=35.964, *P*=3·10^–^6^****^, R^2^=0.814	0.049^*^	2·10^–^7^****^
Anterior lateral ventricle size at 2 weeks post-MCAO^a^	F(2,14)=6.068, *P*=0.013^*^, R^2^=0.388	0.033^*^	0.051
**Locomotive behavior at 10 days post-MCAO**			
Average velocity^a^	F(2,12)=2.158, *P*=0.152, R^2^=–0.126	0.057	0.778
Total distance traveled^a^	F(2,14)=2.105, *P*=0.159, R^2^=0.121	0.059	0.772
**Immunofluorescence at 2 weeks post-MCAO**			
Astrocytosis (GFAP immunoreactivity)^a^	F(2,12)=0.903, *P*=0.429, R^2^=–0.013	0.699	0.231
Microgliosis (Iba1 immunoreactivity)^a^	F(2,12)=5.355, *P*=0.022^*^, R^2^=0.384	0.064	0.051
Mitochondria density (MTCO1 immunoreactivity)^a^	F(2,14)=–0.634, *P*=0.545, R^2^=–0.048	0.410	0.399
Lipid peroxidation (MDA immunoreactivity)^a^	F(2,12)=14.87, *P*=0.001^***^, R^2^=0.665	0.66	0.0002^***^
Cell proliferation (Ki67 immunoreactivity)^a^	F(2,12)=9.282, *P*=0.004^**^, R^2^=0.542	0.418	0.002^**^
Neuronal progenitors (nestin immunoreactivity)^a^	F(2,12)=0.399, *P*=0.680, R^2^=–0.094	0.635	0.526
Histone H3-acetylation (accHH3 immunoreactivity)^a^	F(2,12)=9.136, *P*=0.004^**^, R^2^=0.538	0.649	0.001^***^
Apoptosis (appoptosin immunoreactivity)^a^	F(2,12)=7.661, *P*=0.007^**^, R^2^=0.488	0.942	0.002^**^

**Notes:** Values are mean ± standard error of mean. Significance level *, **, ***, and ****; *P*⩽0.05, 0.01, 0.001, and 0.00001, respectively.

a Measurement of the peri-infarct area ipsilateral to the stroke lesion at 2 weeks post-middle-cerebral artery occlusion (MCAO), expressed as a percentage of the same measure from the corresponding area contralateral to the stroke.

**Table 2:** Infarct volume at 24 hours, 1, and 2 weeks post middle-cerebral artery occlusion (MCAO)

Measurement	Control	LITA
**Volumes by MRI**		
Infarct volume at 24 hours post-MCAO (mm^3^)	130.0±41.6	94.5±35.7
Infarct volume at 1 week post-MCAO (mm^3^)	24.1±10.1	16.1±6.1
Infarct volume at 2 weeks post-MCAO (mm^3^)	58.7±28.6	15.7±7.6
Anterior lateral ventricle volume^a^	177.2±11.90	135.3±10.9*
**Locomotive behavior at 10 days post-MCAO**		
Average velocity (cm/s) at 10 days post-MCAO	15.94±0.57	18.64±1.29*
Total distance traveled (cm) at 10 days post-MCAO	4,559.4±166.8	5,325.3±370.0
**Immunofluorescence at 2 weeks post-MCAO**		
GFAP immunoreactivity^a^	1,088.7±691.7	952.5±367.2
Iba1 immunoreactivity^a^	2,134.3±842.5	1,319.4±444.8*
Nestin immunoreactivity^a^	1.50±0.38	1.18±0.27
MTCO1 immunoreactivity^a^	189.08±24.07	165.23±19.16
Ki67 immunoreactivity^a^	3.64±1.13	2.11±0.45
Appoptosin immunoreactivity score	2.24±0.37	2.21±0.38
MDA immunoreactivity score	2.36±0.52	1.70±0.67
Acetylated-H3 immunoreactivity score	1.68±0.47	1.55±0.54

**Notes:** Percent of anterior lateral ventricle of ipsilateral relative to the contralateral region to the stroke, immunofluorescence measurements at 2 weeks post-MCAO in rats treated with and without liposomal encapsulated acetate (LITA). Values are mean ± standard error of mean;

* *P*⩽0.05.

a Measurement of the peri-infarct area ipsilateral to the stroke lesion, expressed as a percentage of the same measure from the corresponding area contralateral to the stroke.
