# Supplementary figures and images for: Intraperitoneal delivery of acetate-encapsulated liposomal nanoparticles for neuroprotection of the penumbra in a rat model of ischemic stroke
Source: Int J Nanomedicine. 2019 Mar 18;14:1979–91. doi: 10.2147/IJN.S193965 (PMC6430000; doi:10.2147/IJN.S193965)

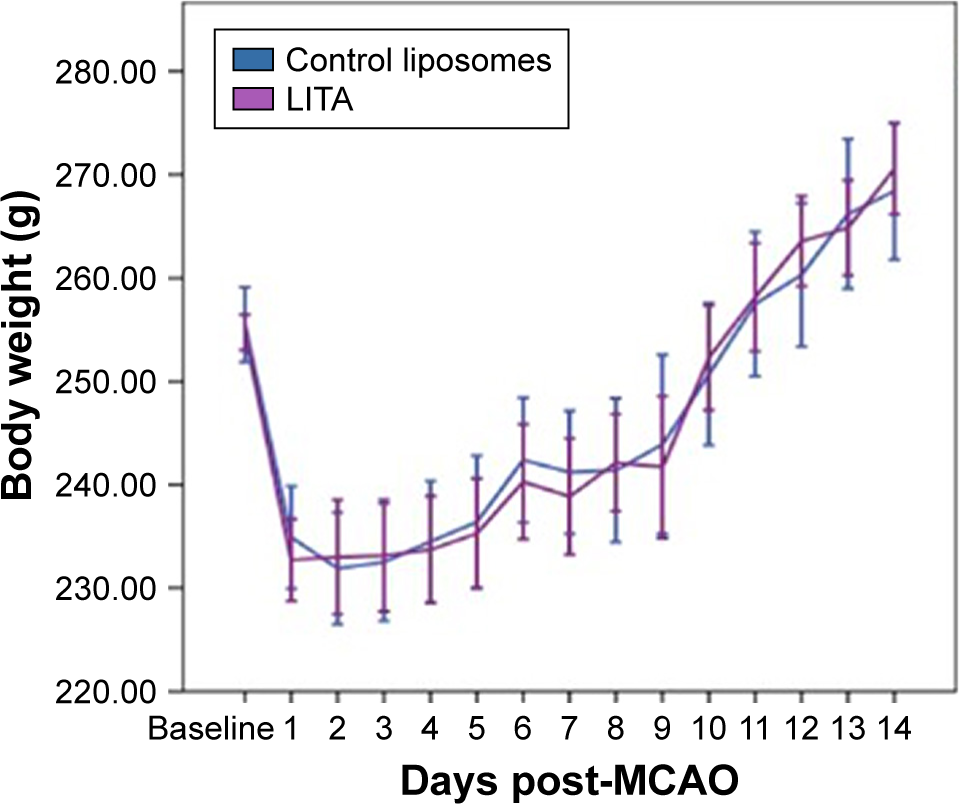

Supplement: Figure S1 — Daily body weights of rats treated with control and liposome-encapsulated acetate (LITA) during the 2 weeks after middle-cerebral artery occlusion (MCAO). [file ijn-14-1979s1.tif]

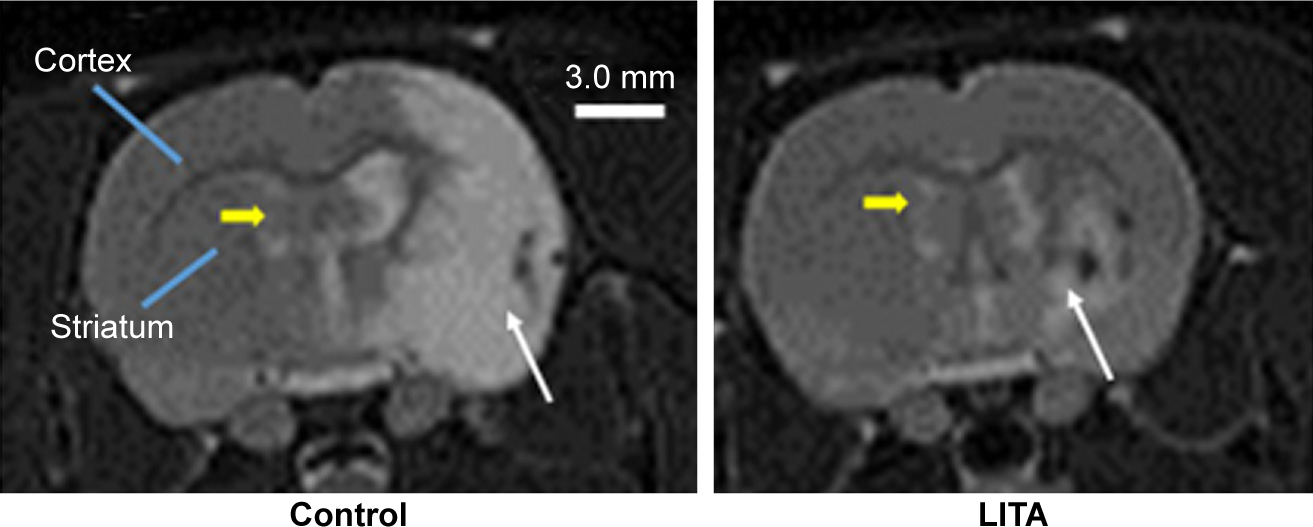

Supplement: Figure S2 — Typical coronal in vivo T2-weighted magnetic resonance images of the brain at −0.10 Bregma of control and liposomal encapsulated acetate (LITA) treated rats at 2 weeks after middle-cerebral artery occlusion. Notes: White and yellow arrows indicate the infarct area and anterior lateral ventricle, respectively. Scale bar: 3.0 mm. [file ijn-14-1979s2.tif]

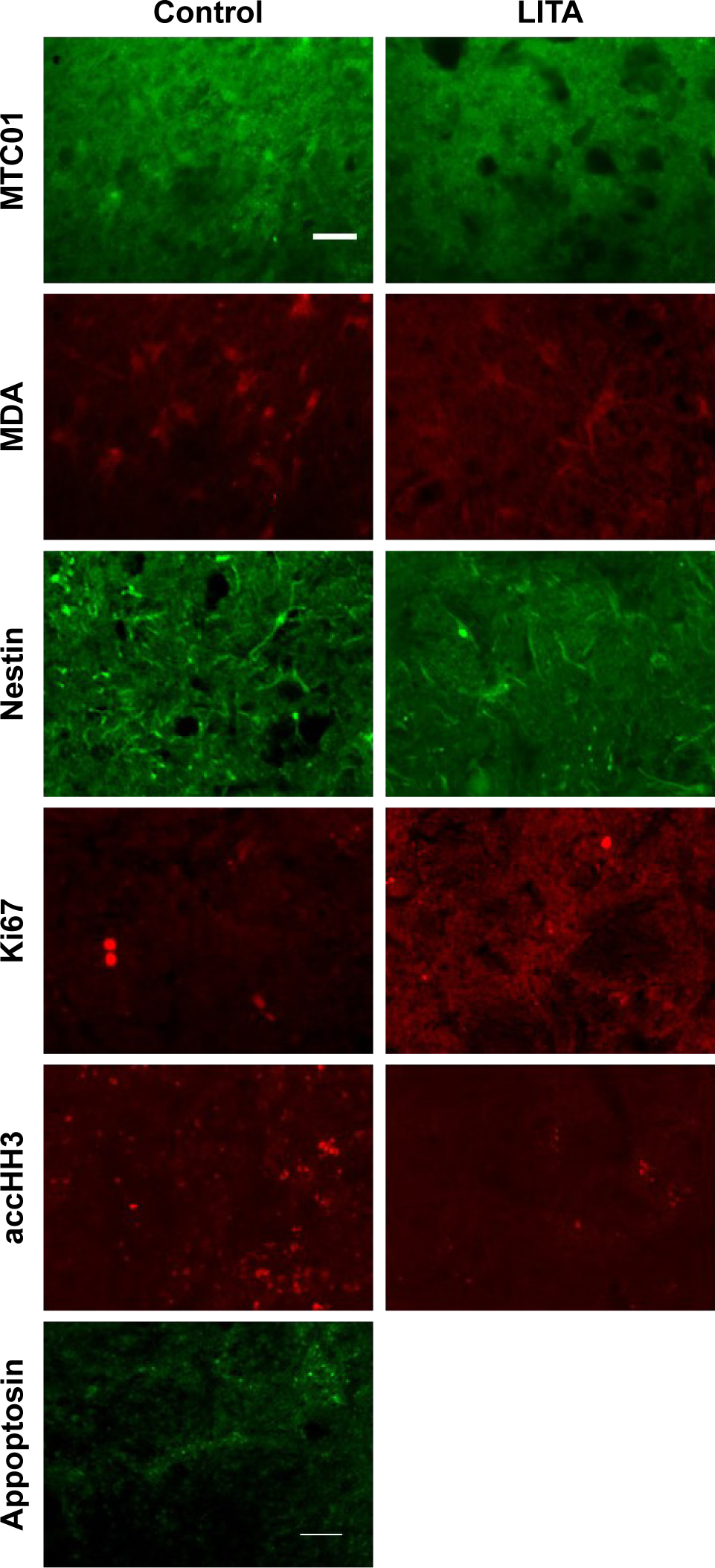

Supplement: Figure S3 — Immunofluorescence for mitochondrial density (MTCO1), lipid peroxidation (malondialdehyde, MDA), neural progenitors (nestin), proliferation (Ki67), histone H3 acetylation (accHH3), and appoptosis (appoptosin) in control or liposomal encapsulated acetate (LITA)-treated animals at 2 weeks after middle-cerebral artery occlusion. Note: Scale bar: 50 micrometer. [file ijn-14-1979s3.tif]
